# Cluster-mediated assembly enables step-growth copolymerization from binary nanoparticle mixtures with rationally designed architectures†
†Electronic supplementary information (ESI) available. See DOI: 10.1039/c8sc00220g


**DOI:** 10.1039/c8sc00220g

**Published:** 2018-04-02

**Authors:** Xianfeng Zhang, Longfei Lv, Guanhong Wu, Dong Yang, Angang Dong

**Affiliations:** a State Key Laboratory of Molecular Engineering of Polymers , Department of Macromolecular Science , Fudan University , Shanghai 200433 , China; b iChem , Shanghai Key Laboratory of Molecular Catalysis and Innovative Materials , Department of Chemistry , Fudan University , Shanghai 200433 , China . Email: agdong@fudan.edu.cn

## Abstract

Directed co-assembly of binary nanoparticles (NPs) into one-dimensional copolymer-like chains is fascinating but challenging in the realm of material science. While many strategies have been developed to induce the polymerization of NPs, it remains a grand challenge to produce colloidal copolymers with widely tailored compositions and precisely controlled architectures. Herein we report a robust colloidal polymerization strategy, which enables the growth of sophisticated NP chains with elaborately designed structures. By quantifying NP assembly statistics and kinetics, we establish that the linear assembly of colloidal NPs, with the assistance of PbSO_4_ clusters, follows a step-growth polymerization mechanism, and on the basis of this, we design and fabricate NP chains structurally analogous to random, block, and alternating copolymers, respectively. Our studies offer mechanistic insights into cluster-mediated colloidal polymerization, paving the way toward the rational synthesis of colloidal copolymers with quantitatively predicted architectures and functionalities.

## Abstract 2

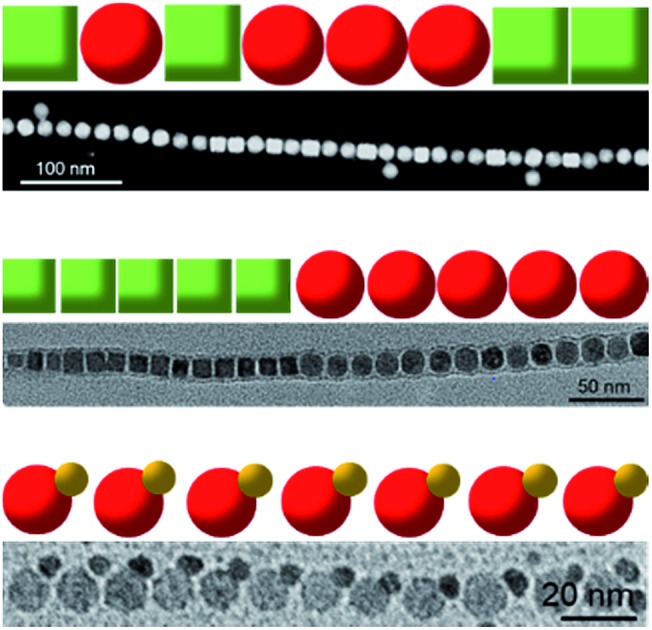
Multicomponent nanoparticle chains structurally analogous to random, block, and alternating copolymers, respectively, have been fabricated by a cluster-mediated self-assembly process.

## Introduction

In spite of the inherent difference between nanoparticles (NPs) and atoms, the do bear resemblance to each other in many ways, especially when NPs self-assemble into ordered structures such as superlattices.^1^–^4^ Co-assembly of two types of NPs into binary superstructures provides a low cost yet efficient way to design metamaterials with tailored properties that arise from the organization and interactions of the constituent NP components.^3^,^4^ Many self-assembly techniques, including solvent-evaporation-induced assembly at solid–2,5 or liquid–air surfaces,^6^,^7^ have been applied to build binary NP superlattices (BNSLs) with three-dimensional (3D) or 2D structures for multiple applications.^8^ In comparison with their 3D and 2D counterparts, the fabrication of 1D binary superstructures, with widely tuned NP components and precisely controlled architectures, is fascinating but more complicated because of the lack of definitive directional interactions between isotropic NPs. Fortunately, the analogy between NP linear assembly and molecular polymerization has led to the concept of colloidal polymerization with NPs behaving like monomers,^9^ enabling the design and growth of chain-like superstructures (denoted as colloidal polymers) based on a molecular approach. Although the forces that govern the assembly and linkage of NP monomers are dramatically different in nature from the case of molecular polymerization, the assembly kinetics and structural features of NP chains can be described by borrowing the knowledge learned in polymer chemistry.^2^,^9^,^10^ Over the past few years, a number of strategies have been proposed to initiate colloidal polymerization, resulting in a variety of 1D NP superstructures with tailored compositions, structures, and functionalities.^9^–^15^

With the development of colloidal polymerization, one would naturally expect that more complicated NP chains reminiscent of copolymers are also obtainable by exploiting the concept of molecular copolymerization. Indeed, many groups have reported the growth of colloid copolymers by co-assembly of binary NPs.^16^–^23^ Existing colloidal copolymerization methods typically rely on dipole-16 or ligand-directed assembly with multiple driving forces (*e.g.*, electrostatic and hydrophobic interactions).^17^–^23^ For instance, Chen and co-workers have demonstrated the linear assembly of Au NPs with different sizes by adjusting the dipole moment of Au NPs with the addition of salts.^16^ Kumacheva and co-workers have reported the step-growth copolymerization of block copolymers comprising polystyrene-functionalized Au and Pt nanorods.^21^ Apart from the limited adjustability of NP compositions (normally noble metal NPs)^16^–^23^ and tedious surface functionalization procedures,^16^–^23^ the colloidal copolymers resulting from these methods usually suffer from low polymerization degrees^16^–^21^ and poor structure control.^16^,^19^–^21^ Moreover, it is still challenging to quantitatively predict the structure of the resulting colloidal copolymers. Therefore, it is highly desirable to develop generalized colloidal copolymerization strategies that enable binary NP chains with widely tailored compositions and rationally designed architectures.

In a previous study, we have shown that colloidal NPs can assemble linearly in solution with the assistance of PbSO_4_ clusters.^24^ As has been reported previously, molecular clusters ligated with long-chain ligands such as oleylamine and octylamine themselves tend to self-assemble into lamellar mesostructures.^13^,^25^ With the addition of NPs capped by the similar long-chain ligands, we have found that these ultrasmall clusters have the tendency to co-assemble with the added NPs, presumably due to the strong van der Waals interactions between the two components.^24^ This co-assembly process, driven by the minimization of the system energy, leads to the stepwise end-on attachment of NPs, resulting in polymer-like chains encapsulated by a half-cylindrical PbSO_4_ cluster shell.^24^ Although this cluster-mediated strategy has been demonstrated to be capable of inducing the linear assembly of a variety of NPs including metals, metal oxides, semiconductors,^24^,^26^ the assembly kinetics and its application to the controlled assembly of binary NP systems have yet to be established.

In this study, by quantifying NP chain growth statistics and kinetics, we establish that this cluster-mediated self-assembly process follows the modes consistent with molecular step-growth polymerization. The validation of the polymerization characteristic of cluster-mediated assembly allows us to apply the synthetic strategies developed in molecular polymerization to the growth of colloidal copolymers. High-quality NP chains with structures analogous to those of random, block, and alternating molecular copolymers, respectively, have been realized by optimizing assembly conditions. This work not only offers mechanistic insights into cluster-mediated assembly, but also opens a step-growth copolymerization route for constructing 1D sophisticated NP superstructures with quantitatively predicted architectures.

## Results and discussion

**Fig. 1:**
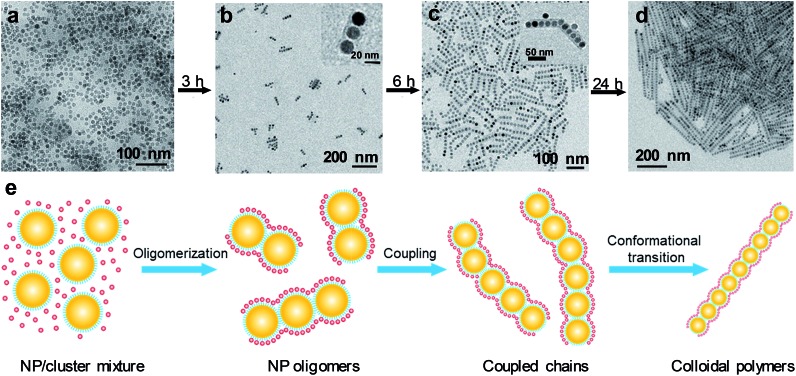
(a–d) TEM images of Fe_3_O_4_ NP ensembles captured at different time intervals. Inset in (b) shows an oligomer consisting of three NPs. Inset in (c) shows a kinked NP chain resulting from oligomer coupling. (e) Schematic illustration of the chain growth process.

Monodisperse Fe_3_O_4_ NPs,^27^ capped with oleic acid, are chosen as the model monomer system to illustrate colloidal polymerization. The self-assembly of polymer chains is triggered by simply mixing Fe_3_O_4_ NPs with the freshly prepared, oleylamine-ligated PbSO_4_ clusters in hexane followed by incubation under ambient conditions for a certain period of time.^24^ To gain insights into the chain growth kinetics, we monitor the temporal evolution of NP assemblies by *ex situ* transmission electron microscopy (TEM). Fig. 1a–d show a series of TEM images captured at different time intervals based on a sample with an initial monomer concentration of [*M*]_0_ = 2.0 × 10^–7^ M (the details for calculating Fe_3_O_4_ NP molar concentrations were provided in ESI†).

Starting with NP and cluster random mixtures (Fig. 1a), we observe the emergence of oligomers consisting of 2 to 6 NPs after 3 h of incubation (Fig. 1b), and only a few free monomers are observable at this stage, suggesting that most NPs have been assembled into oligomers. PbSO_4_ cluster shells that encapsulate NP arrays are also discernible in TEM (Fig. 1b, inset), consistent with previous observations.^24^ As time elapses, these oligomers gradually evolve into longer chains (Fig. 1c) and finally grow into linear polymers consisting of a batch of closed-packed NPs after 24 h of incubation (Fig. 1d). Presumably, individual chains are grown by end-to-end coupling of NP oligomers, as many coupled chains are found to have a kinked morphology (Fig. 1c, inset and S1†). These kinked chain intermediates are expected to undergo nonlinear-to-linear conformational transition during the subsequent chain growth process, considering the nearly straight morphology exhibited by the final polymer chains. A schematic illustrating the chain growth process is given in Fig. 1e. It should be noted that chain branching is not observed during the entire assembly process. We attribute this to the encapsulation effect of the cluster shell,^24^ which essentially inhibits the side-on attachment of NP monomers or oligomers during incubation.

The disappearance of NP monomers in the early assembly stage and the subsequent oligomer coupling events are the key signatures of reaction-controlled step-growth polymerization.^9^,^23^ In order to verify this assumption, a quantitative analysis of chain growth kinetics is desirable. For molecular step-growth polymerization, the reaction of monomers with two functional groups proceeds *via* second-order kinetics when the functional group reactivity is independent of the chain length, which can be described by:^9^,^23^

1 *X*_*n*_ = 4*k*[*M*]*t* + 1

where *X*_*n*_ is the number-average degree of polymerization, *k* is the reaction rate constant, [*M*] is the monomer concentration, and *t* is the reaction time. Likewise, the reaction kinetics of colloidal polymerization can also be described using the same equation. Here, *X*_*n*_ is defined by:^9^,^23^

**Fig. 2:**
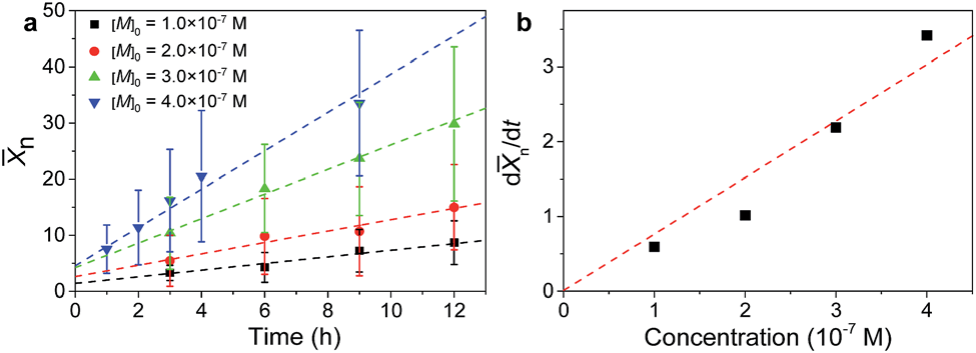
(a) Dependence of the degree of polymerization (*X*_*n*_) on assembly time at different monomer concentrations. For each data point, about 200 chains were counted for statistical analysis. Error bars indicate the standard deviation. The dashed lines are the respective linear fit to the experimental data. (b) Dependence of the polymerization rate (d*X*_*n*_/d*t*) on the monomer concentration. The dashed line is the linear fit to the experimental data.

2 *X*_*n*_ = ∑*n*_*i*_*X*_*i*_/∑*n*_*i*_

where *X*_*i*_ is the number of NPs constituting a single chain and *n*_*i*_ is the number of polymer chains containing *X*_*i*_ NPs. To analyze cluster-mediated assembly kinetics, four samples with different monomer concentrations were examined, and for each sample, aliquots of NP assemblies were taken out the incubation solution at specified time intervals. For each aliquot, the *X*_*n*_ value, determined by statistical analysis, was plotted as a function of the assembly time. As shown in Fig. 2a, the *X*_*n*_ value increases linearly with the assembly time within the explored time range regardless of the initial monomer concentrations, which is characteristic of molecular step-growth polymerization.^9^,^23^ Also evident from Fig. 2a is that the chain growth rate, d*X*_*n*_/d*t*, increases monotonically with the increased monomer concentration, which is also consistent with step-growth polymerization.^9^,^21^,^23^ Plotting the chain growth rate as a function of the monomer concentration yielded a straight line after fitting (Fig. 2b), from which the reaction rate constant *k* was determined to be 6.5 × 10^2^ M^–1^ s^–1^.

It is noteworthy that the polymerization rate constant can be modulated by varying the amount of the free ligands existing in the incubation solution. It has been found that chain growth can be accomplished within 30 min when a small amount of oleylamine is introduced prior to self-assembly.^23^ The increased polymerization rate constant could be attributed to the stronger interactions between PbSO_4_ clusters and Fe_3_O_4_ NPs brought by the added oleylamine ligands, which facilitate the co-assembly of the two components. We also note that the linear assembly of other types of NPs (*i.e.*, metals and semiconductors) should also follow the modes for step-growth polymerization, as this cluster-mediated assembly process, which is independent of NP composition, size, and shape, is mainly dictated by the van der Waals interactions between the ligands (*i.e.*, oleylamine and oleic acid) stabilizing PbSO_4_ clusters and NPs.^24^

Having established the step-growth polymerization characteristic of cluster-mediated assembly, we proceed to the fabrication of sophisticated colloidal copolymers. The microstructure of a molecular copolymer comprising monomers *i* and *j* can be characterized by a microheterogeneity coefficient, *K*, which is defined by:^21^,^28^

**Fig. 3:**
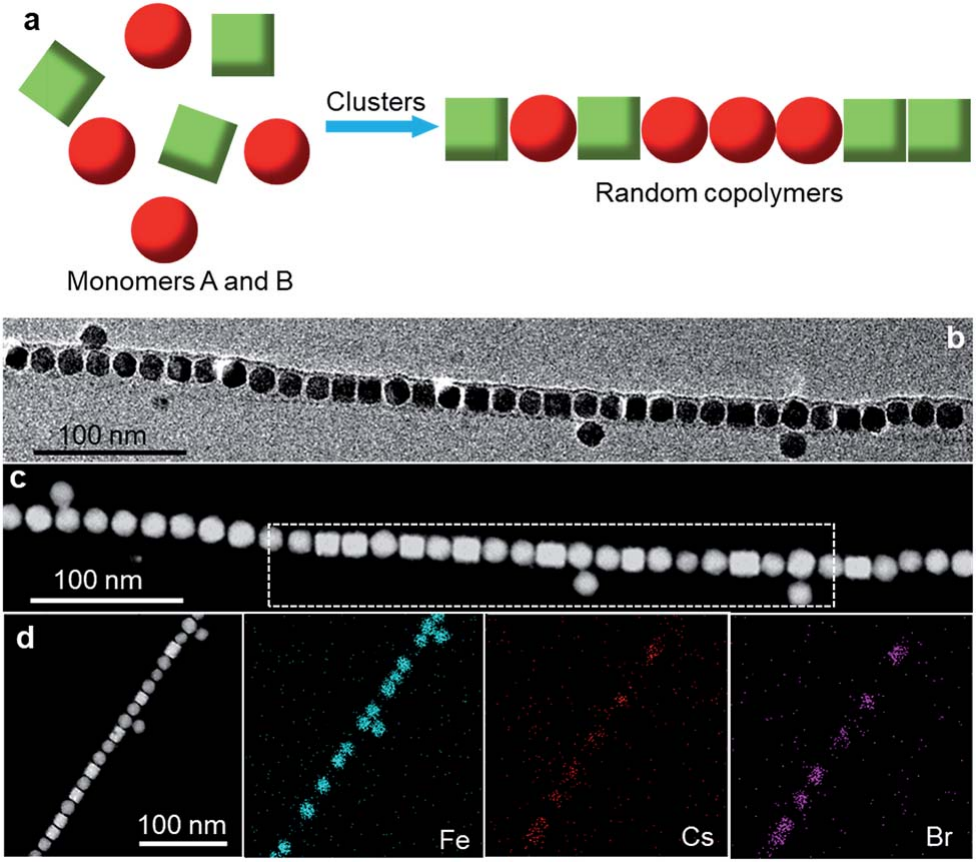
(a) Illustration of the synthesis of random copolymers. For clarity, the NP surface-coating ligands and clusters were omitted. (b) TEM and (c) the corresponding HAADF-STEM images of a hybrid chain self-assembled from 16 nm Fe_3_O_4_ NPs and 15 nm CsPbBr_3_ nanocubes. (d) HAADF-STEM image and the corresponding elemental mapping of the segment indicated by the rectangle in (c).

3 *K* = *P*_*ij*_/(*P*_*ij*_ + 2*P*_*ii*_) + *P*_*ij*_/(*P*_*ij*_ + 2*P*_*jj*_)

where *P*_*ij*_, *P*_*ii*_, and *P*_*jj*_ are the fractions of the pairwise monomer units *i*–*j*, *i*–*i*, and *j–j*, respectively. For molecular copolymers, a *K* value corresponding to block, random, and alternating copolymers is 0, 1, and 2, respectively.^21^,^28^ In order to test the feasibility of cluster-mediated assembly for growing copolymers, we begin with the fabrication of random copolymers by simply co-assembling two NP monomers, namely 16 nm Fe_3_O_4_ NPs and 15 nm CsPbBr_3_ nanocubes, with the assistance of PbSO_4_ clusters, as illustrated in Fig. 3a. Fig. 3b and c show the TEM and the corresponding high-angle annular dark-field scanning TEM (HAADF-STEM) images of a typical chain resulting from co-assembly, in which the spherical and cubic NP monomers can be clearly distinguished. Energy-dispersive X-ray spectroscopy (EDS) elemental mapping provides further evidence for the formation of a hybrid chain (Fig. 3d), where the disordered monomer arrangement is indicative of a structure analogous to random copolymers. Statistical analysis gave rise to a *K* value of ∼0.92, further confirming the random copolymer-like structure. The formation of random copolymers by co-assembly of binary NPs is not surprising, given the generality of this cluster-mediated assembly method.^24^

**Fig. 4:**
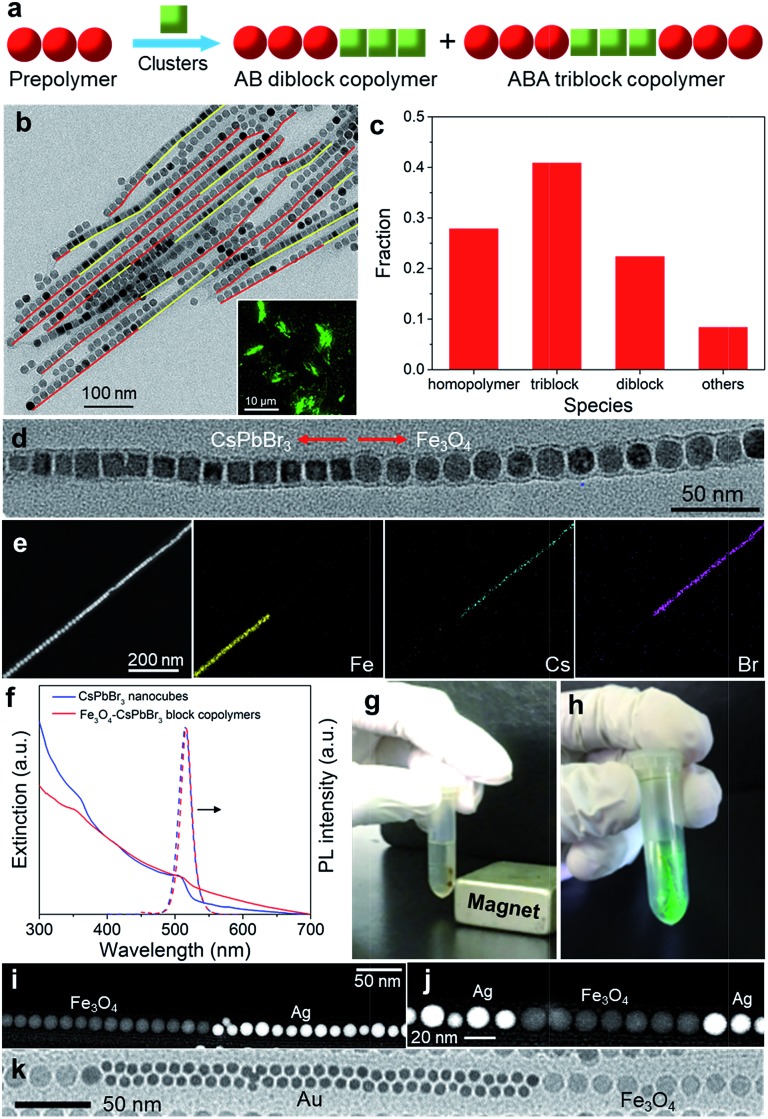
(a) Illustration of the synthesis of block copolymers by the “one-prepolymer” approach. (b) Low-magnification TEM image of Fe_3_O_4_–CsPbBr_3_ block copolymers. For each copolymer chain, the Fe_3_O_4_ and CsPbBr_3_ segments were highlighted in red and yellow, respectively. Inset shows a fluorescence image of Fe_3_O_4_–CsPbBr_3_ block copolymers under UV irradiation. (c) Distribution of the species resulting from copolymerization. About 500 chains were counted for statistical analysis. Others refer to multiblock chains. (d) Magnified TEM image of a single Fe_3_O_4_–CsPbBr_3_ diblock chain. (e) HAADF-STEM image and the corresponding elemental mapping of another diblock chain. (f) Extinction and PL (dashed) spectra of Fe_3_O_4_–CsPbBr_3_ block copolymers and CsPbBr_3_ nanocubes. (g) Photograph showing the attraction of Fe_3_O_4_–CsPbBr_3_ block copolymers by a magnet. (h) Photograph of the dried Fe_3_O_4_–CsPbBr_3_ block copolymers under UV irradiation. (i and j) HAADF-STEM images of di- and triblock copolymers of Fe_3_O_4_–Ag, respectively. (k) TEM image of Fe_3_O_4_–Au block copolymers.

We next seek to fabricate colloidal block copolymers, which possess greater structural regularity compared with their random counterparts. Considering the resemblance between NP assembly and molecular polymerization, we expect that the prepolymerization technique,^21^ which is conventionally used for molecular block copolymer synthesis, may also be applicable to the growth of colloidal block copolymers. To test this hypothesis, we first investigate the feasibility of the “one-prepolymer” strategy,^21^ in which one NP monomer is assembled to form prepolymers followed by the addition of the second monomer. Specifically, 16 nm Fe_3_O_4_ NPs were selected to prepare prepolymers with desired lengths by incubation for a certain period of time. After that, 15 nm CsPbBr_3_ nanocubes and PbSO_4_ clusters were added into the incubation solution containing the as-prepared Fe_3_O_4_ prepolymers to initiate copolymerization, as illustrated in Fig. 4a. The morphology of the resulting Fe_3_O_4_–CsPbBr_3_ copolymers was examined with TEM, HAADF-STEM, and EDS elemental mapping.

Fig. 4b shows a low-magnification TEM image of the resulting Fe_3_O_4_–CsPbBr_3_ block copolymers, in which the intrachain segments consisting of Fe_3_O_4_ NPs and CsPbBr_3_ nanocubes were highlighted in red and yellow, respectively. Impressively, many chains are several hundreds of nanometers long and display a structure reminiscent of AB diblock or ABA triblock copolymers. Note that similar to molecular copolymerization, the homopolymerization of Fe_3_O_4_ oligomers or CsPbBr_3_ monomers cannot be completely inhibited in this “one-prepolymer” approach. Despite the inherent challenge to achieve a high degree of structural homogeneity, statistical analysis shows that di- and tri-block copolymers constitute the majority (∼65%) of the species (Fig. 4c). The microheterogeneity coefficient *K* was determined to be ∼0.1, thus corroborating the high-yield synthesis of block copolymers. Magnified TEM on a single diblock chain reveals the seamless connection between the two segments (Fig. 4d), while HAADF-STEM and the corresponding elemental mapping show distinct spatial distribution of Fe and Cs/Br segments (Fig. 4e), providing further evidence for the formation of a perfect block copolymer-like structure.

Such Fe_3_O_4_–CsPbBr_3_ block copolymers are highly luminescent under UV excitation (Fig. 4b, inset), with a photoluminescence (PL) spectrum similar to that of CsPbBr_3_ monomers (Fig. 4f). The PL quantum yield is in the range of 45–50%, lower than that (∼65%) of the original CsPbBr_3_ monomers. As PbSO_4_ clusters have been demonstrated to have a negligible influence on the luminescent properties of CsPbBr_3_ NPs,^26^ the decreased fluorescence might be caused by the re-absorption effect or energy transfer processes between two NP monomers. The long tail at longer wavelengths in the extinction spectrum (Fig. 4f) could be caused by light scattering arising from the long polymer chains. In addition to the intriguing optical properties, these Fe_3_O_4_–CsPbBr_3_ block copolymers also display strong magnetic response, such that they can be readily separated from the reaction solution by a magnet (Fig. 4g). Notably, the bright green emission was well retained after separation (Fig. 4h). As isolated Fe_3_O_4_ NPs are known to respond weakly to a magnet, the greater response of Fe_3_O_4_–CsPbBr_3_ block copolymers should be attributed to the collective dipolar interactions of close-packed Fe_3_O_4_ NP segments.

Importantly, this “one-prepolymer” strategy can be readily extended to the growth of colloidal block copolymers of diverse compositions by simply selecting desired NP monomers for copolymerization. For instance, Fig. 4i and j show the HAADF-STEM images of Fe_3_O_4_–Ag di- and triblock copolymers, respectively, which are obtained by co-polymerization of 16 nm Ag monomers with Fe_3_O_4_ prepolymers. Likewise, co-polymerization of 8 nm Au monomers with the same Fe_3_O_4_ prepolymers also leads to block copolymers (Fig. 4k). Interestingly, unlike the aforementioned Fe_3_O_4_–CsPbBr_3_ and Fe_3_O_4_–Ag copolymers, the Au segments in Fe_3_O_4_–Au copolymers are composed of double lines of closed-packed Au NPs, probably due to the size-matching effect exerted by the cluster shell, that is, the effective width of two lines of small Au NPs is comparable to that of a single line of larger Fe_3_O_4_ NPs.

In addition to the “one-prepolymer” strategy, we have also attempted to grow colloidal block copolymers by the “two-prepolymer” approach.^21^ In this approach, block copolymers are realized by a coupling reaction between two independently prepared prepolymers (Fig. S2a†). A typical example was shown in Fig. S2b and c,† where Fe_3_O_4_–Ag block copolymers were obtained by incubating 16 nm Fe_3_O_4_ and 16 nm Ag prepolymers in the presence of PbSO_4_ clusters. Although block copolymers can be grown by this approach, most prepolymers remain uncoupled after incubation. The low-yield synthesis could be attributed to the relatively low reactivity between distinct NP chains.

**Fig. 5:**
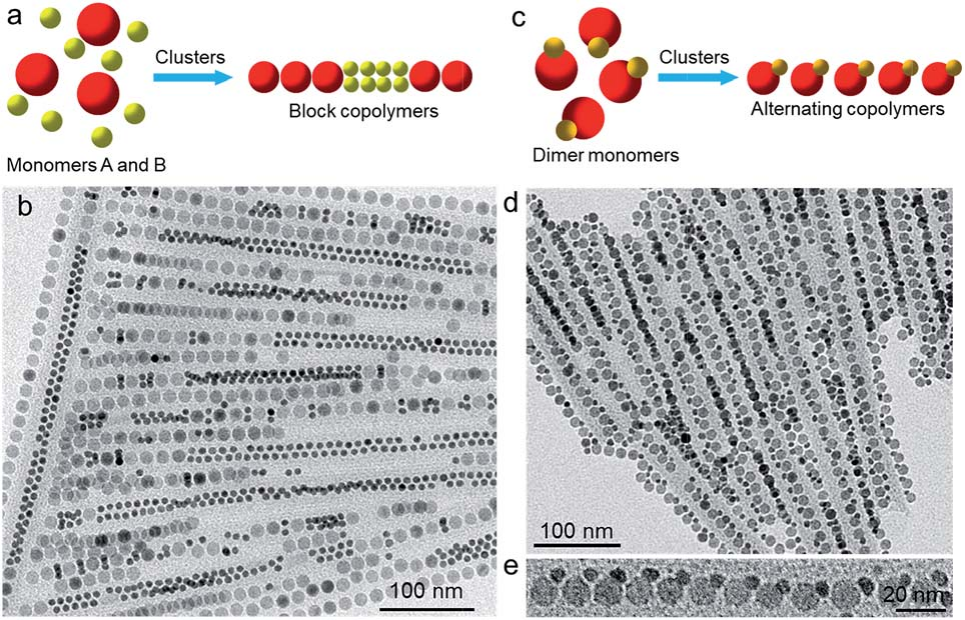
(a) Illustration of the synthesis of block copolymers by co-assembly of binary NPs of distinct sizes. (b) Typical TEM image of Fe_3_O_4_–Au block copolymers resulting from direct co-assembly. (c) Illustration of the synthesis of alternating copolymers by the self-assembly of NP heterodimers. (d) TEM image of alternating copolymer-like assemblies made from Ag–Fe_3_O_4_ NP heterodimers. (e) Magnified TEM image of a perfect alternating chain segment.

It is interesting to note that in addition to the stepwise approaches, colloidal block copolymers can also be achieved in high yield by “one-pot” synthesis by exploiting the intrinsic self-assembly behaviors of colloidal NPs. It is found that co-assembling two types of NPs of distinct sizes often leads to block copolymer-like chains rather than random copolymers, as illustrated in Fig. 5a. Fig. 5b shows a typical TEM image of block copolymers obtained by incubating 16 nm Fe_3_O_4_ and 8 nm Au NPs in the presence of PbSO_4_ clusters, following the procedure used for growing random copolymers. Similar to the Fe_3_O_4_–Au block copolymers synthesized by the “one-prepolymer” approach (Fig. 4k), the copolymers grown by this approach also contain Au-blocks composed of two lines of Au NPs. The evolution of block copolymer-like structures under this situation is attributed to a depletion-induced phase separation process,^29^ where the larger Fe_3_O_4_ NPs tend to assemble while excluding the smaller Au NPs. Co-assembly of 16 nm Fe_3_O_4_ and 6 nm CoFe_2_O_4_ NPs under similar conditions also leads to block copolymers (Fig. S3†), further demonstrating the generality of the approach.

Finally, we extend this cluster-mediated assembly method to the fabrication of alternating copolymers. Compared with random and block copolymers, alternating copolymers are more difficult to achieve due to the challenge to inhibit homopolymerization, and to our knowledge, the assembly of binary alternating NP chains has been rarely explored.^17^,^18^ One conceptually straightforward, albeit experimentally challenging, way to achieve alternating copolymers is to engineer the structure of NP building blocks. Fortunately, recent advances in colloidal synthesis allow us to synthesize a wide range of NPs, among which heterogeneous dimers^30^ could be a promising candidate for building NP chains structurally analogous to alternating copolymers by exploiting shape-directed assembly (Fig. 5c). Fig. 5d shows a low-magnification TEM image of such an example, where the chains, self-assembled from Ag–Fe_3_O_4_ NP heterodimers (Fig. S4†),^31^ show an alternating copolymer-like structure (Fig. 5e). We should note that these NP chains are conceptually different from real alternating copolymers, as there is only one actual monomer. Nonetheless, the microheterogeneity coefficient *K* was determined to be ∼1.77, only slightly deviating from the value (*i.e.*, 2) expected for an ideal alternating structure, confirming the formation of an alternate copolymer-like structure. Presumably, the small *K* value is caused by the presence of a fraction of imperfect alternating chains. Further modification of the dimer structure by tuning the size and/or shape of the two components could help to improve the quality of alternating copolymer-like chains.

The inclined alignment of Ag–Fe_3_O_4_ dimers in chains can be explained by the templating effect of PbSO_4_ cluster shells. As we and others have demonstrated previously,^24^,^26^ the half-cylindrical PbSO_4_ cluster shells, having a width dimension of ∼30–40 nm, behave like a self-template to confine the assembly of NPs. Our Ag–Fe_3_O_4_ dimers have an overall size of ∼28 nm (considering the presence of organic ligands), close to the width of the cluster shells. As a consequence, Ag–Fe_3_O_4_ dimers will tend to assemble with certain inclination within PbSO_4_ cluster shells. In this context, the close packing of Ag–Fe_3_O_4_ dimers can proceed *via* either AB–AB or AB–BA modes (Fig. S5†), yet we mainly observe the AB–AB configuration in the resulting assemblies (Fig. 5e). We suspect that the preferential AB–AB alignment is energetically more favourable compared with the AB–BA mode. However, further studies are needed to elucidate the underlying mechanism.

## Conclusions

In summary, we have verified a mechanism of step-growth polymerization for the 1D cluster-mediated NP assembly process. Based on guidelines in macromolecular chemistry, colloidal copolymers made up of binary NP mixtures with structures reminiscent of those of random, block, and alternating copolymers, respectively, have been designed and fabricated. Apart from the robust process, the generality of this copolymerization strategy allows the ready tuning of NP combinations. This provides unique possibilities for tailoring the properties of these multifunctional colloidal copolymers. This work not only offers mechanistic insights into cluster-mediated colloidal polymerization, but also paves the way toward the synthesis of colloidal copolymers with quantitatively predicted architectures and functionalities.

## Conflicts of interest

There are no conflicts to declare.

## Supplementary Material

Supplementary information

## References

[cit1] Nai J., Guan B., Yu L., Lou X. (2017). Sci. Adv..

[cit2] Kim J., Ou Z., Jones M. R., Song X., Chen Q. (2017). Nat. Commun..

[cit3] Redl F. X., Cho K. S., Murray C. B., O'Brien S. (2003). Nature.

[cit4] Talapin D. V. (2008). ACS Nano.

[cit5] Shevchenko E. V., Talapin D. V., Kotov N. A., O'Brien S., Murray C. B. (2006). Nature.

[cit6] Dong A., Chen J., Vora P. M., Kikkawa J. M., Murray C. B. (2010). Nature.

[cit7] Dong A., Ye X., Chen J., Murray C. B. (2011). Nano Lett..

[cit8] Boles M. A., Engel M., Talapin D. V. (2016). Chem. Rev..

[cit9] Liu K., Nie Z., Zhao N., Li W., Rubinstein M., Kumacheva E. (2010). Science.

[cit10] Wang H., Chen L., Shen X., Zhu L., He J., Chen H. (2012). Angew. Chem., Int. Ed..

[cit11] Klinkova A., Therien-Aubin H., Ahmed A., Nykypanchuk D., Choueiri R. M., Gagnon B., Muntyanu A., Gang O., Walker G. C., Kumacheva E. (2014). Nano Lett..

[cit12] Sutter E., Sutter P., Tkachenko A. V., Krahne R., de Graaf J., Arciniegas M., Manna L. (2016). Nat. Commun..

[cit13] Jana S., Davidson P., Abécassis B. (2016). Angew. Chem., Int. Ed..

[cit14] Zhuang Z., Jiang T., Lin J., Gao L., Yang C., Wang L., Cai C. (2016). Angew. Chem., Int. Ed..

[cit15] Luo B., Smith J. W., Ou Z., Chen Q. (2017). Acc. Chem. Res..

[cit16] Yang M., Chen G., Zhao Y., Silber G., Wang Y., Xing S., Han Y., Chen H. (2010). Phys. Chem. Chem. Phys..

[cit17] Zhang S., Kou X., Yang Z., Shi Q., Stucky G. D., Sun L., Wang J., Yan C. (2007). Chem. Commun..

[cit18] Zhao Y., Xu L., Liz-Marzan L. M., Kuang H., Ma W., Asenjo-Garcia A., Garcia de Abajo F. J., Kotov N. A., Wang L., Xu C. (2013). J. Phys. Chem. Lett..

[cit19] Xia H., Su G., Wang D. (2013). Angew. Chem., Int. Ed..

[cit20] Klinkova A., Therien-Aubin H., Choueiri R. M., Rubinstein M., Kumacheva E. (2013). Proc.
Natl. Acad. Sci..

[cit21] Liu K., Lukach A., Sugikawa K., Chung S., Vickery J., Therien-Aubin H., Yang B., Rubinstein M., Kumacheva E. (2014). Angew. Chem., Int. Ed..

[cit22] Li W., Kanyo I., Kuo C. H., Thanneeru S., He J. (2015). Nanoscale.

[cit23] Luo B., Smith J. W., Wu Z., Kim J., Ou Z., Chen Q. (2017). ACS Nano.

[cit24] Zhang X., Lv L., Ji L., Guo G., Liu L., Han D., Wang B., Tu Y., Hu J., Yang D., Dong A. (2016). J. Am. Chem. Soc..

[cit25] Wang Y., Liu Y., Zhang Y., Kowalski P. J., Rohrs H. W., Buhro W. E. (2013). Inorg. Chem..

[cit26] Pan A., Jurow M., Zhao Y., Qiu F., Liu Y., Yang J., Urban J. J., He L., Liu Y. (2017). Nanoscale.

[cit27] Park J., An K., Hwang Y., Park J. G., Noh H. J., Kim J. Y., Park J. H., Hwang N. M., Hyeon T. (2004). Nat. Mater..

[cit28] Kuchanov S., Slot H., Stroeks A. (2004). Prog. Polym. Sci..

[cit29] Sikorski M., Sandy A. R., Narayanan S. (2011). Phys. Rev. Lett..

[cit30] Zhu H., Nagaoka Y., Hills-Kimball K., Tan R., Yu L., Fang Y., Wang K., Li R., Wang Z., Chen O. (2017). J. Am. Chem. Soc..

[cit31] Zhang L., Dou Y. H., Gu H. C. (2006). J. Colloid Interface Sci..

