# Supplementary material for: Cluster-mediated assembly enables step-growth copolymerization from binary nanoparticle mixtures with rationally designed architectures
Source: Chem Sci. 2018 Apr 2;9(16):3986–91. doi: 10.1039/c8sc00220g (PMC5944820; doi:10.1039/c8sc00220g)
Supplement: Supplementary file 1 [file SC-009-C8SC00220G-s001.pdf]

Electronic Supplementary Material (ESI) for Chemical Science.
This journal is © The Royal Society of Chemistry 2018

Supplementary Information

Cluster-mediated assembly enables step-growth copolymerization from binary nanoparticle mixtures with rationally designed architectures

Xianfeng Zhang,[‡a] Longfei Lv,[‡b] Guanhong Wu,[b] Dong Yang[a] and Angang Dong*[b]

[a]*State Key Laboratory of Molecular Engineering of Polymers and Department of Macromolecular Science, Fudan University, Shanghai 200433, China.*
[b]*iChem, Shanghai Key Laboratory of Molecular Catalysis and Innovative Materials, and Department of Chemistry, Fudan University, Shanghai 200433, China. Email: agdong@fudan.edu.cn.*
[‡]*These authors contributed equally to this work.*

Experimental Section

Materials: Oleic acid (OA, 90%), oleylamine (OAm, 70%), 1-octadecene (ODE, 90%), benzyl ether (98%), cobalt(II) acetylacetonate ($Co(acac)_2$, 97%), iron(III) acetylacetonate ($Fe(acac)_3$, 97%), lead(II) bromide ($PbBr_2$, 99.999% metals basis), and tetrabutylammonium hydrogen sulfate (TBAHS, 97.0%) were purchased from Sigma-Aldrich. Sodium oleate (98.0%), iron chloride hexahydrate ($FeCl_3 \cdot 6H_2O$), borane-tert-butylamine complex (TBAB, 95.0%), silver nitrate ($AgNO_3$, 99.8%), and lead chloride ($PbCl_2$, 99.999%) were purchased from Aladdin. Hydrogen tetrachloroaurate(III) trihydrate ($HAuCl_4 \cdot 4H_2O$, 99.99%) and cesium carbonate (Cs_2CO_3, 99.994% metals basis) were purchased from Alfa Aesar. 1,2-hexadecanediol (98.0%) was purchased from TCI. Silver acetate (99.0%) was purchased from Amethyst. OA, OAm, and ODE were dried under vacuum at 120 °C for 1 h and stored in an Ar-purged glovebox. Other materials were used as received without further purification.

Synthesis of Fe_3O_4 NPs: Monodisperse Fe_3O_4 NPs with a diameter of ~16 nm were synthesized according to a modified literature method.[1] Typically, 36 g of iron oleate (pre-synthesized by reaction of $FeCl_3 \cdot 6H_2O$ and sodium oleate) and 8.6 g of OA were dissolved in 150 g of ODE, followed by degassing at 120 ^{o}C for 60 min. The resulting solution was then heated to 320 ^{o}C under N_2 atmosphere for 1 h. After cooling down to room temperature, the crude solution was purified by centrifugation with the addition of methanol and isopropanol for three times. The sediments were redispersed in 40 mL of hexane for further use.

Synthesis of $CoFe_2O_4$ NPs: $CoFe_2O_4$ NPs with a diameter of ~6 nm were synthesized by a modified literature method.[2] Generally, 5.6 g of $Co(acac)_2$, 2.0 g of $Fe(acac)_3$, 0.65 g of 1,2-hexadecanediol, 21.0 g of OAm, and 4.5 g of OA were added to 25 mL of benzyl ether in a three-neck flask. The solution was degassed on a Schlenk line under vacuum and subsequently heated, under nitrogen, at 110 ^{o}C for 1 h and 200 ^{o}C for 2 h. After that, the solution was heated to 295 ^{o}C and kept at this temperature for 1 h.

$CoFe_2O_4$ NPs were separated from the growth solution using ethanol followed by centrifugation and were redispersed in 20 mL of hexane.

Synthesis of Au NPs: Monodisperse Au NPs with a diameter of ~8 nm were synthesized by a modified literature method.[3] Typically, 100 mg of $HAuCl_4{\cdot}4H_2O$, 10 mL of OAm, and 10 mL of hexane were combined in air and magnetically stirred at 15 ^oC under N_2 flow for 10 min. Then, a reducing solution containing 15 mg of TBAB, 1 mL of OAm, and 1 mL of hexane was quickly injected. The reaction mixture was allowed to react at 15 ^oC for 1 h before 60 mL of ethanol was added to precipitate Au NPs. The precipitated Au NPs upon centrifugation were redispersed in 10 mL of hexane.

Synthesis of Ag NPs: Colloidal Ag NPs with a diameter of ~16 nm were synthesized by modifying a reported approach.[4] Briefly, 170 mg of $AgNO_3$ and 20 mL of dried OAm were heated at 60 ^oC under N_2. After that, the mixture was quickly heated up to 240 ^oC and maintained at this temperature for 1 h. Acetone was added to precipitate and purify Ag NPs. The precipitated Ag NPs upon centrifugation were redispersed in 15 mL of hexane.

Synthesis of $CsPbBr_3$ nanocubes: To synthesize cesium oleate, 0.814 g of Cs_2CO_3, 40 mL of ODE, and 2.5 mL of OA were mixed and dried under 120 ^oC for 1 h, and then heated under N_2 to 150 ^oC until all Cs_2CO_3 was dissolved. $CsPbBr_3$ nanocubes with a diameter of ~15 nm were synthesized according to the protocol reported previously.[5] In a typical synthesis, a mixture of 5 mL of ODE and 0.69 mg of $PbBr_2$ was dried under vacuum at 120 ^oC for 1 h, followed by the injection of 0.5 mL of dried OA at 120 ^oC. After the dissolution of $PbBr_2$, the temperature was raised to 200 ^oC and the stock solution containing cesium oleate was quickly injected. The reaction mixture was quickly cooled by an ice-water bath. The crude solution was centrifuged, and the sediment was redispersed in 10 mL of hexane for further use.

Synthesis of Ag-Fe_3O_4 heterodimers: The synthesis of Ag-Fe_3O_4 heterodimers was carried out based on a procedure reported previously.[6] Typically, 50 mg of 16 nm Fe_3O_4 NPs, 50 mg of silver acetate, and 3 mL of OAm were added into 40 mL of toluene. The mixture was heated to 80 ^{o}C under N_2 and kept at this temperature for 8 h. After cooling down to room temperature, the stock solution was centrifuged with the addition of ethanol. The precipitated Ag-Fe_3O_4 dimers upon centrifugation were redispersed in 15 mL of hexane.

Synthesis of $PbSO_4$ clusters: $PbSO_4$ clusters were synthesized by the protocol we reported previously.[7] In brief, lead oleate was synthesized by heating a mixture of 139 mg of $PbCl_2$ and 8 mL of dried OA up to 150 ^{o}C under N_2 to form a clear solution. In a separate vial, 4.3 mg of TBAHS and 0.4 mL of OAm were added into 8 mL of toluene under sonication. After that, 0.4 mL of the stock solution containing lead oleate was added into this solution under continuous stirring for 5 min. The resulting solution was centrifuged with the addition of 10 mL of ethanol. The resulting white precipitate was redispersed in 5 mL of chloroform to form a clear colorless solution.

Cluster-mediated polymerization of colloidal NPs into homopolymers: Inorganic homopolymers were obtained by a cluster-mediated assembly process, following a modified procedure reported previously.[7] To initiate colloidal polymerization, an appropriate amount of the freshly made $PbSO_4$ cluster solution was added into a hexane solution containing NPs followed by incubation for a certain period of time under ambient conditions. The resulting inorganic polymers were isolated by centrifugation followed by redispersion in hexane for further characterization.

Co-assembly of binary NPs into random copolymers: The procedure for growing random copolymers was conducted similarly to that used for the synthesis of inorganic homopolymers, except that a mixture containing two types of NPs with similar sizes were co-incubated in the presence of $PbSO_4$ clusters.

Synthesis of inorganic block copolymers by the stepwise copolymerization approaches: Inorganic block copolymers could be synthesized by the prepolymerization strategy commonly used in molecular copolymerization. In the first “one-prepolymer” approach, block copolymers were synthesized by co-polymerization of NP prepolymers with a second NP monomer with the assistance of $PbSO_4$ clusters. Take Fe_3O_4-$CsPbBr_3$ block copolymers for example, 16 nm Fe_3O_4 NPs were first allowed to polymerize to afford prepolymers with desired lengths by incubation for a certain period of time. After that, an appropriate amount of 15 nm $CsPbBr_3$ nanocubes and $PbSO_4$ clusters were added into the solution containing the as-prepared Fe_3O_4 prepolymers to initiate copolymerization. The resulting copolymer species were isolated by centrifugation and were then re-dispersed in hexane for further characterization. Block copolymers could also be obtained by the “two-prepolymer” approach, in which two kinds of prepolymers with desired lengths were coupled with the assistance of $PbSO_4$ clusters. It should be noted that the yield of block copolymers was much lower than that obtained by the “one-prepolymer” approach.

“One-pot” synthesis of inorganic block copolymers: In this approach, inorganic block copolymers were formed by the direct assembly of binary NPs with distinct sizes, following the procedure used for growing random copolymers. The evolution of block copolymers under this situation was attributed to a depletion-induced phase separation process, where the larger NPs tend to assemble while excluding the smaller ones.

Fabrication of inorganic alternating copolymer-like assemblies: To achieve alternating copolymer-like assemblies, Ag-Fe_3O_4 heterodimers were used as monomers, which were incubated with the presence of $PbSO_4$ clusters.

Characterization: Transmission electron microscopy (TEM), high-resolution TEM (HRTEM), and high-angle annular dark-field scanning TEM (HAADF-STEM) images and energy dispersive X-ray spectroscopy (EDS) elemental mapping were collected using a Tecnai G2 F20 S-TWIN microscope operated at 200 kV. UV-visible extinction

spectra were carried out on a Shimadzu UV-3600 UV-vis-NIR spectrophotometer. Steady-state photoluminescence (PL) spectra were obtained at room temperature on an Edinburgh Instruments FLS920 fluorescence spectrophotometer. The absolute PL quantum yield was obtained using integrating sphere measurements. Fluorescence micrographs were obtained on a Leica DM4000 B LED microscope equipped with a Leica DFC310 FX camera.

Calculation of Fe_3O_4 monomer molar concentrations: To calculate the monomer molar concentrations (M), the mass concentration (mg mL^{-1}) of Fe_3O_4 NPs was first determined by completely drying the solvent hexane. Take the Fe_3O_4 monomer solution with a molar concentration of 2.0×10^{-7} M for example, the measured mass concentration is 1.63 mg mL^{-1}. The mass of a single Fe_3O_4 NP (density = 5.18 g/cm^3) with a diameter of 16 nm is $(4\pi/3)\times(8\times10^{-7})^3\times5.18 = 1.11\times10^{-17}$ g. Assuming that the surface-coating oleic acid ligands constitute 15 wt% of the total mass of a single Fe_3O_4 NP,[8] the number of Fe_3O_4 NPs per mL of the incubation solution should be $1.63\times0.85\times10^{-3}/1.11\times10^{-17} = 1.25\times10^{14}$, corresponding to ~$2.0\times10^{-10}$ mol. As a result, the molar concentration of Fe_3O_4 NP monomers is 2.0×10^{-7} M.

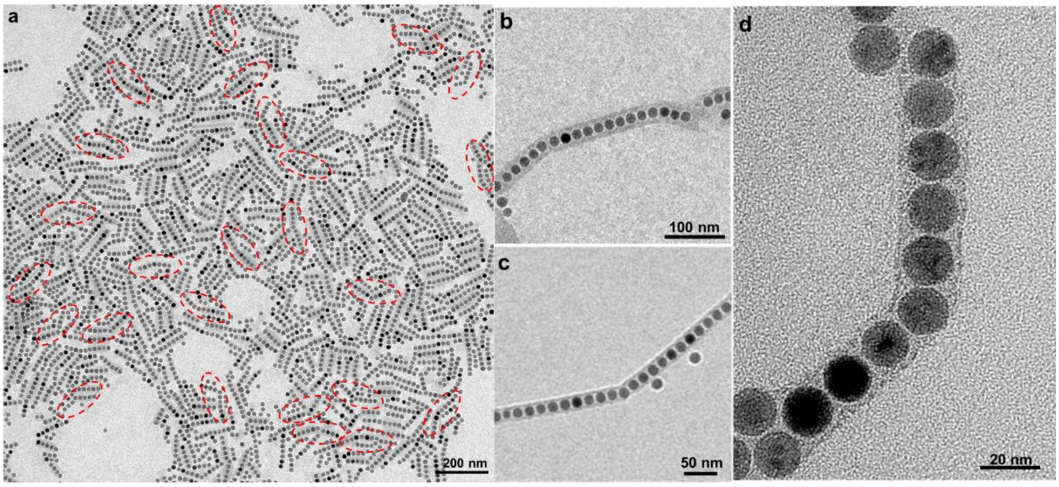

Fig. S1. (a) Low-magnification TEM image of Fe_3O_4 NP chains resulting from oligomer coupling, showing that a number of chains (indicated by the dashed ellipses) have a kinked morphology. (b, c) High-magnification TEM images of kinked Fe_3O_4 NP chains. (d) HRTEM image of a single kinked chain.

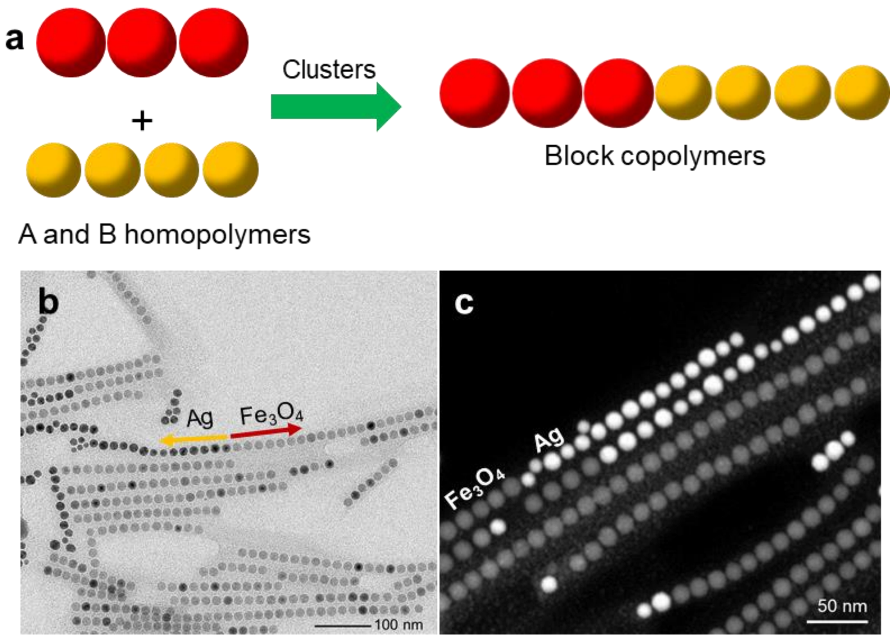

Fig. S2. (a) Schematic illustrating the synthesis of block copolymers by the "two-prepolymer" strategy. (b) Typical TEM image of the species resulting from the coupling of 16 nm Fe_3O_4 and 16 nm Ag prepolymers, showing that only a few block copolymers can be formed by this approach. (c) HAADF-STEM image of Fe_3O_4-Ag block copolymers grown by this "two-prepolymer" strategy.

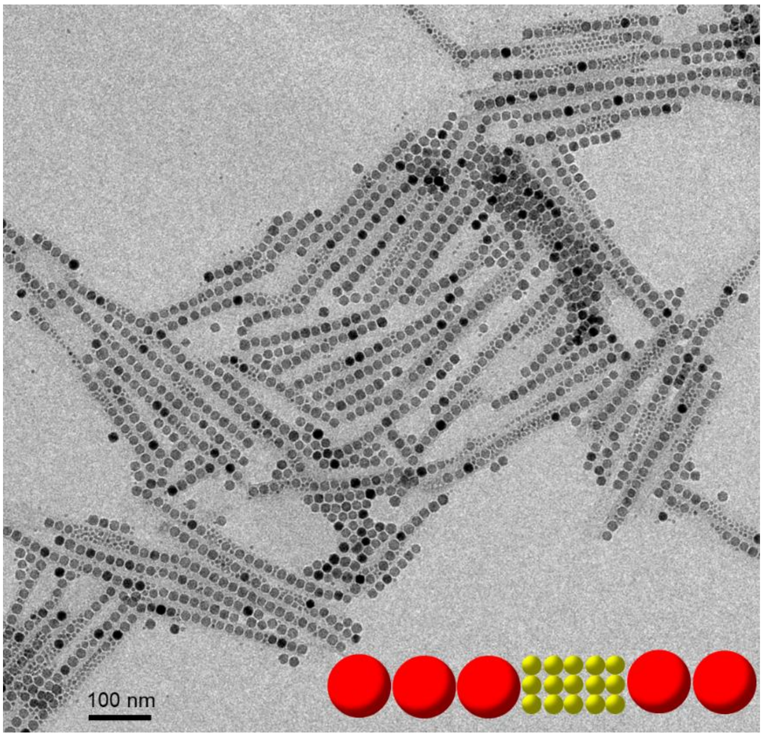

Fig. S3. Typical TEM image of Fe_3O_4-$CoFe_2O_4$ block copolymers obtained by direct co-assembly of 16 nm Fe_3O_4 and 6 nm $CoFe_2O_4$ NPs. In many cases, the $CoFe_2O_4$-blocks were composed of three lines of $CoFe_2O_4$ NPs (as schematically shown in the inset), due to the size-matching effect.

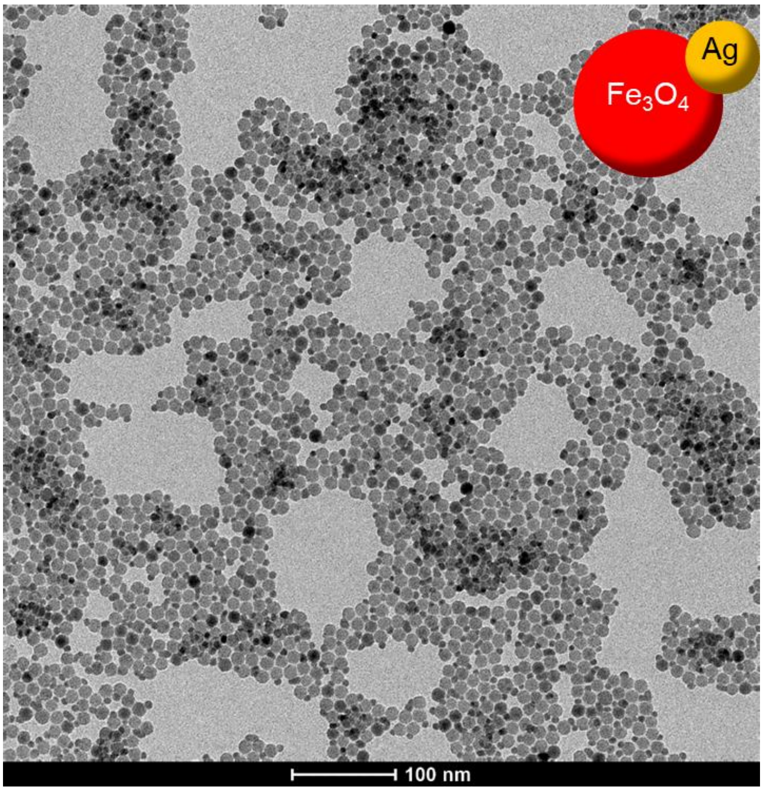

Fig. S4. Typical TEM image of dumbbell-like Ag-Fe_3O_4 NP heterodimers used for the growth of alternating copolymers. The size of Ag and Fe_3O_4 components was 8 and 16 nm, respectively. Inset shows a cartoon illustration of the heterodimer.

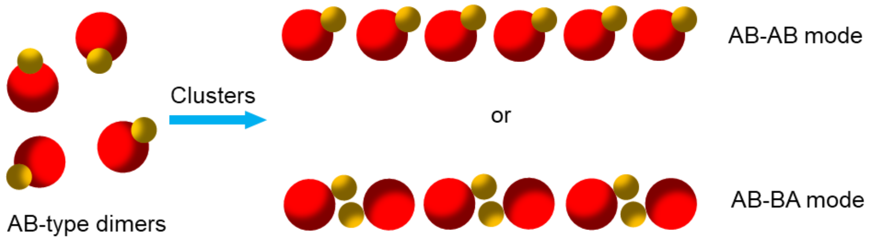

Fig. S5. Illustration of the possible alignment modes of heterodimers during the formation of alternate copolymer-like structures.

References

[1] J. Park, K. An, Y. Hwang, J. G. Park, H. J. Noh, J. Y. Kim, J. H. Park, N. M. Hwang, T. Hyeon, *Nat. Mater.* **2004**, *3,* 891-895.

[2] A. L. Strickler, M. Escudero-Escribano, T. F. Jaramillo, *Nano Lett.* **2017**, *17*, 6040-6046.

[3] B. Wu, H. Yang, H. Huang, G. Chen, N. Zheng, *Chin. Chem. Lett.* **2013**, *24*, 457-462.

[4] S. Peng, J. M. McMahon, G. C. Schatz, S. K. Gray, Y. Sun, *Proc. Natl. Acad. Sci.* **2010**, *107*, 14530-14534.

[5] L. Protesescu, S. Yakunin, M. I. Bodnarchuk, F. Krieg, R. Caputo, C. H. Hendon, R. Yang, A. Walsh, M. V. Kovalenko, *Nano Lett.* **2015**, *15*, 3692-3696.

[6] L. Zhang, Y. H. Dou, H. C. Gu, *J. Colloid Interf. Sci.* **2006**, *297*, 660-664.

[7] X. Zhang, L. Lv, L. Ji, G. Guo, L. Liu, D. Han, B. Wang, Y. Tu, J. Hu, D. Yang, A. Dong, *J. Am. Chem. Soc.* **2016**, *138*, 3290-3293.

[8] Y. Jiao, D. Han, Y. Ding, X. Zhang, G. Guo, J. Hu, D. Yang, A. Dong, *Nat. Commun.*, **2015**, *6*, 6420.
